# Interplay among manures, vegetable types, and tetracycline resistance genes in rhizosphere microbiome

**DOI:** 10.3389/fmicb.2024.1392789

**Published:** 2024-07-01

**Authors:** Izhar Ali, Beenish Naz, Ziyang Liu, Jingwei Chen, Zi Yang, Kotb Attia, Nasir Ayub, Ikram Ali, Arif Ahmed Mohammed, Shah Faisal, Likun Sun, Sa Xiao, Shuyan Chen

**Affiliations:** ^1^Key Laboratory of Cell Activities and Stress Adaptations Ministry of Education, School of Life Sciences, Lanzhou University, Lanzhou, Gansu, China; ^2^State Key Laboratory of Herbage Improvement and Grassland Agroecosystems, College of Ecology, Lanzhou University, Lanzhou, Gansu, China; ^3^Department of Biochemistry, College of Science, King Saud University, Riyadh, Saudi Arabia; ^4^Korean Environmental Microorganism Resource Center, Department of Integrative Biotechnology, Sungkyuankwan University, Seoul, Republic of Korea; ^5^Center for Chinese Herbal Medicine Drug Development, Hong Kong Baptist University, Kowloon Tong, China; ^6^Department of Environmental Engineering, School of Architecture and Civil Engineering, Chengdu University, Chengdu, China; ^7^College of Animal Sciences, Gansu Agricultural University, Lanzhou, Gansu, China

**Keywords:** tetracycline resistance genes, manure fertilization, plant microbes interaction, rhizosphere, mobile genetic elements, risk of exposure

## Abstract

The rapid global emergence of antibiotic resistance genes (ARGs) is a substantial public health concern. Livestock manure serves as a key reservoir for tetracycline resistance genes (TRGs), serving as a means of their transmission to soil and vegetables upon utilization as a fertilizer, consequently posing a risk to human health. The dynamics and transfer of TRGs among microorganisms in vegetables and fauna are being investigated. However, the impact of different vegetable species on acquisition of TRGs from various manure sources remains unclear. This study investigated the rhizospheres of three vegetables (carrots, tomatoes, and cucumbers) grown with chicken, sheep, and pig manure to assess TRGs and bacterial community compositions via qPCR and high-throughput sequencing techniques. Our findings revealed that tomatoes exhibited the highest accumulation of TRGs, followed by cucumbers and carrots. Pig manure resulted in the highest TRG levels, compared to chicken and sheep manure, in that order. Bacterial community analyses revealed distinct effects of manure sources and the selective behavior of individual vegetable species in shaping bacterial communities, explaining 12.2% of TRG variation. Firmicutes had a positive correlation with most TRGs and the *intl1* gene among the dominant phyla. Notably, both the types of vegetables and manures significantly influenced the abundance of the *intl1* gene and soil properties, exhibiting strong correlations with TRGs and elucidating 30% and 17.7% of TRG variance, respectively. Our study delineated vegetables accumulating TRGs from manure-amended soils, resulting in significant risk to human health. Moreover, we elucidated the pivotal roles of bacterial communities, soil characteristics, and the *intl1* gene in TRG fate and dissemination. These insights emphasize the need for integrated strategies to reduce selection pressure and disrupt TRG transmission routes, ultimately curbing the transmission of tetracycline resistance genes to vegetables.

## Graphical Abstract

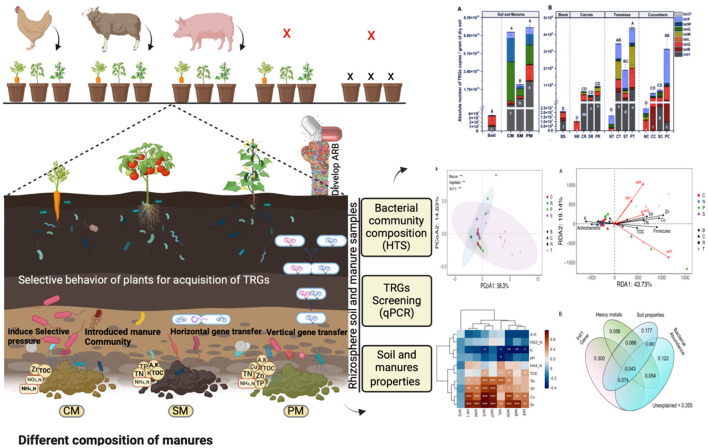

## 1 Introduction

Discovery of antibiotics represents a pivotal historical breakthrough in the treatment of bacterial diseases in both humans and animals (Wang et al., 2021). However, extensive use of antibiotics, particularly tetracycline, and application of heavy metals in livestock and poultry farms, along with the subsequent use of animal manure in agriculture, have become significant pathways for introducing drug residues, heavy metals, resistant bacterial communities, and tetracycline resistance genes (TRGs) into agricultural soils (FAO and WHO, 2019; Marutescu et al., 2022). Tetracycline compounds are frequently detected in various types of manures and in agricultural soils treated with animal manure (Carballo et al., 2016). These agricultural practices significantly increase the likelihood of dissemination of manure-derived TRGs into the environment and the human food chain (Muhammad et al., 2020; Guo et al., 2021; Sorinolu et al., 2021). Recent research has highlighted the interplay of antimicrobial resistance (AMR) between environmental bacteria and human pathogens, highlighting the importance of the One Health approach in addressing this global health concern (Forsberg et al., 2012; Jiang et al., 2017; Sajjad et al., 2020; Kim and Cha, 2021). It is estimated that a lack of immediate action could cause millions of deaths worldwide and cost the world's economy 100 trillion US$ by 2050 (Salam et al., 2023; World Health Organisation, 2023). Thus, immediate action is required to reduce the emerging threat of antimicrobial resistance.

Tetracycline, a broad-spectrum antibiotic, is extensively used in animal husbandry and accounts for a significant proportion of the global consumption of antibiotics used in animal production between 2010 and 2015 (Chang et al., 2023). Recent research indicates that TRGs are the most prevalent antibiotic resistance genes (ARGs) in animal and human microbiomes, constituting over 90% of identified ARGs in animal-associated metagenomes (Pal et al., 2016; Kang et al., 2018). This emphasizes the need to address use of TRGs in animal manures to curb the emergence and dissemination of TRGs and associated bacteria in the environment. While animal manure enhances soil fertility and crop productivity, it also serves as a reservoir for antibiotic-resistant bacteria (Martinez, 2009; Li et al., 2015; Chen et al., 2018). The metabolism of the majority of antibiotics is poor and are eventually excreted into the environment, along with a substantial amount of ARG-carrying bacteria derived from manure (Sarmah et al., 2006). Depending on the manure sources and soil characteristics, bacteria and pathogens from manure can persist in soils for weeks to months (Heuer et al., 2008; Hu et al., 2016; Leclercq et al., 2016).

As an organic fertilizer, manure enriches the soil microbial population, introducing manure-borne microbiomes and facilitating horizontal gene transfer (HGT) of ARGs among them (Smalla et al., 2001; Hu et al., 2016). Additionally, the rhizosphere can fuel its microbiota by providing abundant and readily available energy and carbon sources (Bais et al., 2006; Wallenstein, 2017). The diversity and composition of the rhizosphere bacterial community are influenced by both plant species and soil properties (Garbeva et al., 2008; Vorholt et al., 2017). Plants exert selective effects on rhizobacterial assemblages from bulk soil and bacterial reservoirs in manure to acquire specific functional traits necessary for plant health (Pérez-Jaramillo et al., 2016; Yan et al., 2016; Howard et al., 2020). Plant species or even genotypes tend to assemble relatively distinct rhizobacterial communities (Pérez-Jaramillo et al., 2016; Li et al., 2018). Consequently, the rhizosphere microbiome acquires a distinct pool of TRGs from these manures and soil (Bais et al., 2006; Schreiter et al., 2014; Bai et al., 2015). Plant-associated microbes function as a medium for the interplay between environmental and human microbiomes, potentially transmitting environmental ARGs to humans (Chen et al., 2019).

Manure can stimulate the horizontal transfer of antibiotic resistance genes (ARGs) in soil, and the plant rhizosphere acts as a hub for HGT, potentially transmitting ARGs from the soil to plant bacterial communities (Pu et al., 2020; Wang F. et al., 2022; Wang J. et al., 2022; Wang Y. et al., 2022; Chen et al., 2023). HGT poses significant risks to human health, as ARGs, particularly TRGs, have been detected in vegetables and fruits grown in manure-amended soil (Bulgarelli et al., 2012; Wang F. et al., 2022; Wang J. et al., 2022; Wang Y. et al., 2022). As a result, ARGs can be transferred from manure-amended soil to the rhizosphere soil and then back to the bacterial communities of plants, especially in vegetables that are consumed raw (Berger et al., 2010; Marti et al., 2013; Wang et al., 2015; Vestergaard et al., 2019). Further research is needed to understand how different vegetables acquire TRGs from various types of manure. Hence, the objectives of this study are twofold: first to explore the interaction between manure fertilization and different vegetable species regarding the acquisition and abundance of the tetracycline resistome in rhizosphere soil. Second, to identify the factors driving selection and persistence of TRGs within these distinct ecological niches. The results of this study provide insights that enhance our understanding of how various vegetables contribute to persistence and dissemination of manure-derived TRGs in rhizosphere soil, which will invariably be valuable in developing strategies for mitigation of the spread of TRGs into the food chain, ultimately safeguarding human health.

## 2 Materials and methods

### 2.1 Soil and manure sampling

Chicken manure (CM), sheep manure (SM), pig manure (PM), and non-agricultural soil samples were collected from Hongliutai village, Liujiaxia township of Yongjing county, Gansu province, northwest China (35°47′-36°12′N and 102°53′-103°39′E) in October 2018. Soil samples (taken at a depth of 5–20 cm) that had not been treated with manure for the last 10 years were collected and promptly transported to the laboratory on ice, where the soils were homogenized and divided into three parts. The first part was immediately used for the pot experiment; the second was stored at 4°C for analyzing soil physicochemical properties; and the third was stored at −20°C for DNA extraction to screen for bacterial community structure and TRG profile prior to the pot experiment. The animal manures were collected within 1–2 days of defecation and transported on ice to the laboratory. CM, SM, and PM manures were air-dried in shade until they reached the standard moisture content (< 60%). After that, these manures were carefully mixed with soils to get a final concentration of 80 mg of manure g^−1^ of dry soil, equivalent to a standard rate of 60 m^3^ of manure per hectare (Han et al., 2018). The key characteristics of the manures and soil used in this experiment are presented in Supplementary Table S1. Each collected manure was thoroughly mixed before use.

### 2.2 Greenhouse pot experiment

The greenhouse pot experiment was performed at Lanzhou University in October 2018. A quantity of 84 kg of soil was thoroughly mixed with 4.2 kg of autoclaved vermiculite. The soil was clay-type in nature, so vermiculite was added to maintain favorable conditions for vegetable growth. Thirteen treatment groups were established in plastic pots (23.5 × 14.0 cm, 2-kg pot for tomatoes and cucumbers, and 36 × 21 cm, 3 kg for carrots). The first letter indicates the manure treatments and the second vegetable types in treatment labeling. The treatment groups were as follows: untreated soil group with no manure and no vegetables (BS), carrots (*Daucus carota*) without (NR) chicken, sheep, and pig manures (10 g air-dried manures/kg of soil); CR, SR, and PR, respectively, similarly tomatoes **(*Solanum lycopersicum*)** without (NT) chicken, sheep, and pig manures (10 g air-dried manures/kg of soil); CT, ST, and PT, respectively, and cucumbers (*Cucumis sativus*) without (NC) chicken, sheep, and pig manures (10 g air-dried manures/kg of soil); CC, SC, and PC, respectively. A total of 13 treatments with three replicates were organized in a randomized block design in a greenhouse at Lanzhou University, which was maintained at 25°C in daylight (16 h) and 20°C in dark (8 h) (65% humidity). Deionized water was used to water the plants every second day to maintain 70% of water-holding capacity and harvested at day 90 when all the vegetables reached maturity. Rhizosphere soil samples (2–8 cm depth) were carefully collected in sterile conditions to avoid cross-contamination and transferred into individual sampling bags for analysis.

### 2.3 Soil properties

The rhizosphere soil was sieved using a 100 mesh (0.15 mm) and analyzed for total phosphorus (TP), total nitrogen (TN), soil organic carbon (SOC), ammonium (NH^4+^), nitrate (NO^3−^) content and pH. Total phosphorus (TP) and total nitrogen (TN) were digested by concentrated H_2_SO_4_ and their contents measured by Mo–Sb antispetrophotography and semi-micro Kjeldahl (Tian et al., 2017), respectively, by using an auto-chemistry analyzer (Smart-Chem 200, AMS Alliance, United States). Soil organic carbon was measured using the wet oxidation method (Wang et al., 2018; Naz et al., 2023). After extraction with 2M KCl, ammonium and nitrate levels were measured following the method by Wang J. et al. (2022). Heavy metals were quantified using the previously published technique (Qian et al., 2016). The pH of the soil in a 1:1.5 soil water slurry was measured using a pH meter (PHSJ-3F, Shanghai INESA Scientific Instrument Co., Ltd., China).

### 2.4 DNA extraction and PCR

DNA was extracted from 0.25 g of each sample using the DNeasy R PowerSoil R Kit (Qiagen, Hilden, Germany) following the manufacturer's instructions. The NanoDrop ND2000c spectrophotometer (NanoDrop Technologies, Wilmington, DE, USA) was used to measure the concentration and purity of the extracted DNA. The presence of 10 desired genes (*tetB, tetG, tetL* (efflux genes), *tetM, tetQ, and tetW* (ribosomal protection genes), *tetX and tet37* (an activation gene), *IntI1* (mobile genetic element), and the 16S rRNA gene) for total bacteria was determined by PCR in soil and manure samples. PCR products were analyzed by gel electrophoresis using 1.5% (w/v) agarose in 1 × TBE buffer. Each primer used in this study was previously validated; details are provided in Supplementary Table S2. After being purified using a PCR Fast-Spin PCR Product Purification Kit from Tiangen Biotech in Beijing, China, positive amplification products were ligated into the pMD19-T vector from TaKaRa Biotech Co. Ltd. in Dalian, China. *Escherichia coli* DH5α was used as the bacterial host to amplify recombinant plasmid vectors (TransGen Biotech, Beijing, China) (Wang et al., 2018). Using the BLAST software (http://blast.ncbi.nlm.nih.gov/Blast.cgi), PCR products were sequenced and analyzed for validation.

### 2.5 Quantification of targeted genes

Targeted genes and the 16S rRNA gene were quantified using real-time quantitative PCR with SYBR Green, and the concentration of target genes was measured to their standard curve of isolated plasmid vectors. The Agilent Bioanalyzer 2100 system (Agilent, United States) was used for the detection and analysis. The PCR reaction was performed in 20 μL volume according to the manufacturer's specifications, using 10 μL SYBR Premix Ex Taq II (TaKaRa, Dalian, China), 1 μL DNA template, 0.8 μL primer (10 μM), and 7.4 μL molecular biology grade water (Wang et al., 2018). Product specificity was tested by melting curves, efficiency, R^2^ value, and gel-electrophoresis. qPCR efficiency was kept at 90–120%, and R^2^ values for the standardization curves of each gene were > 99.0%.

### 2.6 Next-generation sequencing of the 16s rRNA gene

The primer pairs 515F (5-GTGCCAGCMGCCGCGGTAA-3) and 806R (5-GGACTACHVGGGTWTCTAAT-3) were used to amplify the V4 hypervariable regions of the bacterial 16S rRNA gene (Caporaso et al., 2011; Khan et al., 2022a; Wang Y. et al., 2022). As per the devised protocol, using 2% agarose gel, the PCR products were visualized and purified using the GeneJET Gel-Extraction Kit (Thermo Scientific). Long S5TM-XL instrument (Thermo Fisher Scientific) was used to sequence the purified 16S rRNA amplicons. Sequenced data were quality-filtered using vsearch-GITHUB (Wang et al., 2012), and Cutadapt. v1.9.1 (Jiao et al., 2017) was employed to perform chimera detection. Using Uparse v7.01, the high-quality sequences were binned and divided into various operational taxonomic units (OTUs; 97% similarity) (Rognes et al., 2016; Khan et al., 2022b). Finally, a total of 81,333 bacterial OTUs were generated, with an average of 2,700 clean reads per sample. The raw nucleotide sequences were submitted to the National Center for Biotechnology Information (NCBI) Sequence Read Archive (SRA) under the BioProject accession number PRJNA1022849. The dada2-paired pipeline built into the QIIME2 program (QIIME2.2021.2) was used to create OTUs with 99% similarity.

### 2.7 Statistical analysis

All data were analyzed using R software v4.0.2 (R Core Team, 2021). Statistical comparisons of soil properties, TRGs, and bacterial communities among different vegetable types and manure soils were conducted using Tukey's HSD and a two-way permutation test. To assess the impact of vegetable species and manure treatments on TRGs and bacterial communities, ordination analyses were performed employing a vegan package (Dixon, 2003) to visualize differences in the TRGs among treatment groups, and a principal coordinates analysis (PCoA) was performed using the Euclidean dissimilarity index. Additionally, PCoA using the Bray–Curtis dissimilarity index was performed to visualize differences in the microbial community composition among treatment groups. Then, permutational multivariate analysis of variance (PERMANOVA) was conducted to assess the effects of various vegetable species and manure treatments on TRGs and bacterial communities. Heat maps were generated using the “pheatmap” package based on Pearson' correlations. Moreover, redundancy analysis (RDA) was carried out to assess the effects of both abiotic (soil properties) and biotic (bacterial dominant phylum) factors on TRGs. The variance in TRGs by the effects of abiotic and biotic factors was calculated through variation partitioning analysis (VPA) based on the RDA. The shared and unique OTUs were also displayed using Venn diagrams created through the Omicshare (version) online software.

## 3 Results

### 3.1 Role of vegetables in shaping soil abiotic properties from different manures

The study findings revealed the significant enhancements observed in targeted soil properties following manure amendments and the notable influence of different vegetable types on soil properties. Among the vegetables, tomatoes (T) exhibited the most prominent impact, followed by cucumbers (C) and carrots (R). However, the influence of vegetables varied across different soil parameters. The effects in manure-amended soils are more apparent than those in blank soil (BS). For instance, compared to (N) and across the manure treatments, the pig manure-treated group (P) resulted in significant enhancements in soil properties, notably increased soil Cu and Zn concentrations, and TN, TOC, and TP, indicating a substantial enrichment of these soil properties and heavy metals (Cu and Zn), which are strongly correlated with most of the TRGs (Figure 4). Chicken manure-treated groups (C) had a notable enhancement in Zn, AK, TN, and NO_3_-N. Sheep manure-treated groups (S) showed significant enhancement in TOC and TN compared to no manure-treated (N) and BS control groups. Interestingly, there were significant associations between manure and vegetable treatments, indicating the role of vegetable types in shaping rhizosphere soil properties from various manures (Table 1). Overall, the results highlight the dynamic interactions between manure sources and vegetable types in shaping soil abiotic properties, emphasizing the importance of considering vegetable types with manures in agricultural practices for optimal soil health and resistome dissemination.

**Table 1:** Effects of different manures, vegetables, and their interaction on soil abiotic properties (mean ± SE).

**Soil properties**	**Cu mg/kg**	**Zn mg/kg**	**K g/kg**	**A.K mg/kg**	**TN mg/L**	**TP mg/L**	**NO3_N mg/L**	**NH4_N mg/L**	**TOC g/kg**
BS	d18.07 ± 0.37	d51.3 ± 0.51	b22,880 ± 400	de128.3 ± 3.7	f0.08 ± 0.008	b0.63 ± 0.02	c0.44 ± 0.18	a0.18 ± 0.04	f4,153 ± 76
NR	d18.9 ± 0.79	d51.4 ± 0.43	ab23,826 ± 353	**c170.6** **±9.2**	**de0.12** **±0.03**	b0.63 ± 0.01	c2.09 ± 0.54	a0.22 ± 0.04	de8,580 ± 693
CR	d21.7 ± 0.58	c66.7 ± 3.9	ab23,261 ± 1,022	**a422.6** **±11**	**a1.84** **±0.34**	ab1.5 ± 0.53	**a22.2** **±2.4**	a0.23 ± 0.02	**a1894** **±1,364**
SR	d20.3 ± 0.43	d54.8 ± 0.68	ab23,553 ± 510	**c174.3** **±5.04**	**bc0.47** **±0.02**	b0.81 ± 0.07	c1.08 ± 0.15	a0.24 ± 0.03	**bc14,300** **±958**
PR	**bc33.6** **±2.06**	c70.4 ± 2.3	ab23,144 ± 157	cde144.6 ± 5.9	**bc0.65** **±0.1**	ab1.2 ± 0.13	c2.42 ± 0.42	a0.23 ± 0.01	**bc13,200** **±842**
NT	d19.8 ± 1.5	cd60 ± 1.05	**a25,118** **±264**	e111.6 ± 4.7	ef0.25 ± 0.23	ab1.18 ± 0.39	c1.62 ± 0.25	a0.23 ± 0.06	ef4,620 ± 660
CT	cd24.7 ± 1	**b83.3** **±0.75**	**b22,508** **±381**	de130 ± 12	**cd0.65** **±0.21**	ab1.15 ± 0.17	**b7.8** **±0.77**	a0.28 ± 0.02	**cd11,000** **±702**
ST	cd23.9 ± 0.77	c67.05 ± 1.9	**b22,875** **±140**	**cd161.6** **±6.9**	**ab1.10** **±0.05**	b0.92 ± 0.01	c1.19 ± 0.18	a0.28 ± 0.03	**ab17,380** **±596**
PT	**a47.2** **±4.01**	**a106.1** **±3.1**	**b22,983** **±93**	de127.3 ± 5.2	**abc1.51** **±0.03**	a1.9 ± 0.16	c0.88 ± 0.01	a0.22 ± 0.006	**abc15,180** **±457**
NC	d18.9 ± 0.31	d52.8 ± 1.1	ab23,422 ± 259	de131.3 ± 3.3	**bc0.13** **±0.08**	b0.70 ± 0.06	c2.09 ± 0.36	a0.14 ± 0.02	**bc14,106** **±1,146**
CC	d21.1 ± 0.6	c70.1 ± 3	b22,871 ± 685	**cd163** **±7.8**	ef0.52 ± 0.08	ab1.01 ± 0.02	c2.4 ± 1.08	a0.25 ± 0.01	ef4,620 ± 1,008
SC	d22.8 ± 0.58	cd60.2 ± 1.1	b23,000 ± 298	**b229.3** **±4.3**	**ab1** **±0.11**	ab1.06 ± 0.31	c2.59 ± 0.47	a0.18 ± 0.02	**ab16,940** **±1,326**
PC	**ab39.1** **±3.7**	**b86.2** **±4.3**	b22,605 ± 306	e123.6 ± 9.2	**ab1** **±0.03**	ab1.53 ± 0.13	c0.94 ± 0.04	a0.18 ± 0.02	**ab16,720** **±850**
Vegetables	**0.0024** ^**^	**< 2e-16** ^***^	0.75	0.072 ‘.'	**0.0008** ^***^	0.058 ‘.'	0.082	0.511	**0.0215** ^*^
Manures	**< 2e-16** ^***^	**< 2e-16** ^***^	**0.0032** ^**^	**< 2.2e-16** ^***^	**< 2.2e-16** ^***^	**< 2.2e-16** ^***^	**< 2.2e-16** ^***^	0.07 ‘.'	**0.0014** ^***^
V^*^M	0.07 ‘.'	**< 2e-16** ^***^	0.13	**< 2.2e-16** ^***^	**< 2.2e-16** ^***^	0.207	**< 2.2e-16** ^***^	0.53	**0.0024** ^***^

The first letters B, N, C, S, and P represented blank, no, chicken, sheep, and pig manures, respectively; the second letters S, R, T, and C represented blank, carrots, tomatoes, and cucumber, respectively. while Cu, Copper; Zn, Zinc; K, total Potassium; AK, Available Potassium; TN, total nitrogen; TP, total phosphorus; NO3_N, Nitrates; NH4_N, Ammonium and; TOC, Total organic carbon. Significant. Codes: 0 ‘^***^' 0.001 ‘^**^' 0.01 ‘^*^' and 0.05 ‘.' (two-way permutation test) and small letters displayed Tukey HSD results.

### 3.2 Influence of vegetables and manures on soil TRGs and *intl1* gene

**Figure 1:**
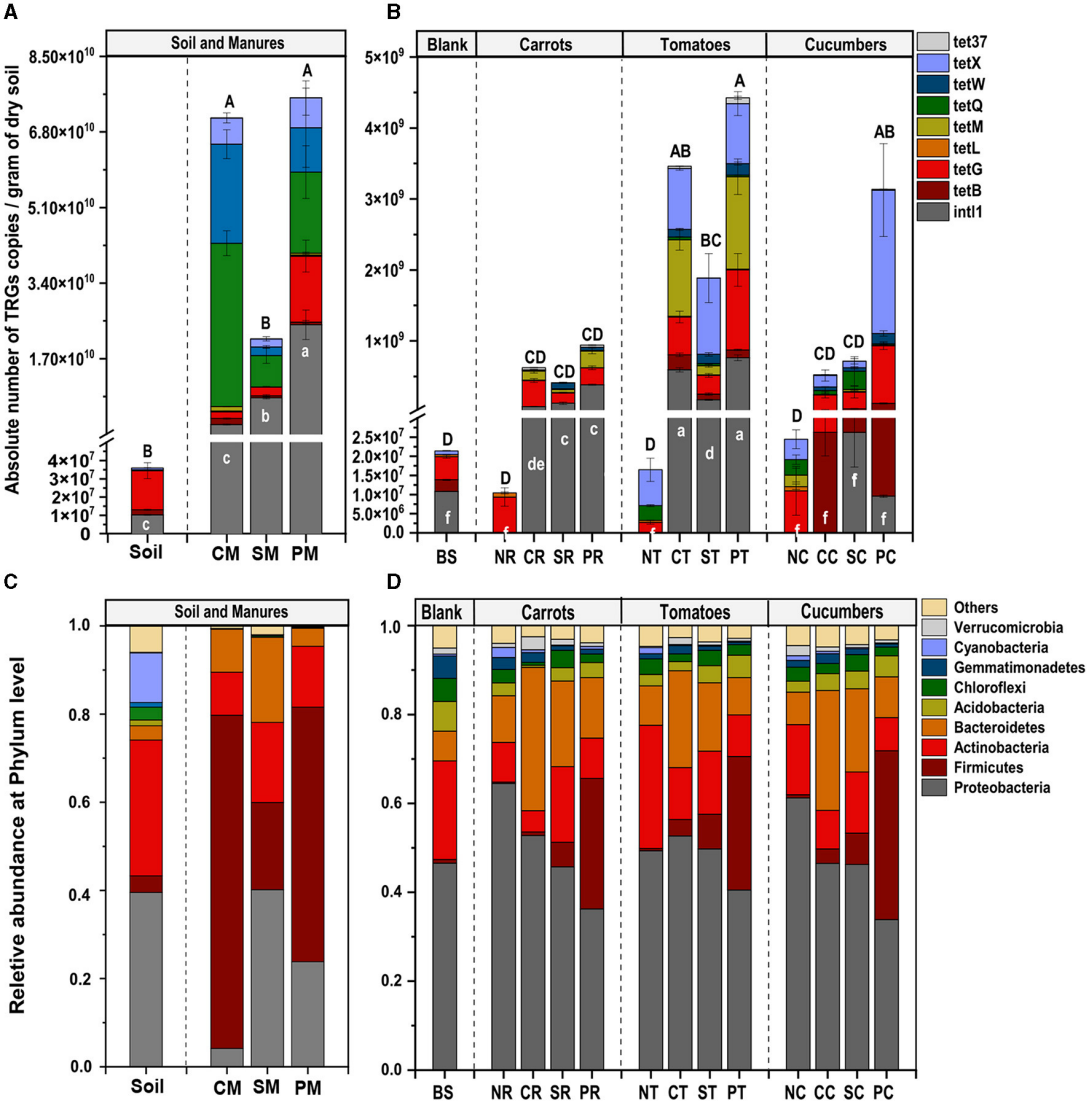
Average number of TRGs and *intl1* gene (**A** before and **B** after treatments) and bacterial community (**C** before and **D** after treatments) in vegetable rhizosphere soil amended with different sources of manures. Different capital letters above the bars indicate the average significant difference in TRGs, and small letters showed a significant difference in *intl1* (*P* < 0.05). the first letters B, N, C, S, and P represented blank, no, chicken, sheep, and pig manures, respectively; the second letters S, R, T, and C represented blank soil, carrots, tomatoes, and cucumber, respectively.

**Figure 2:**
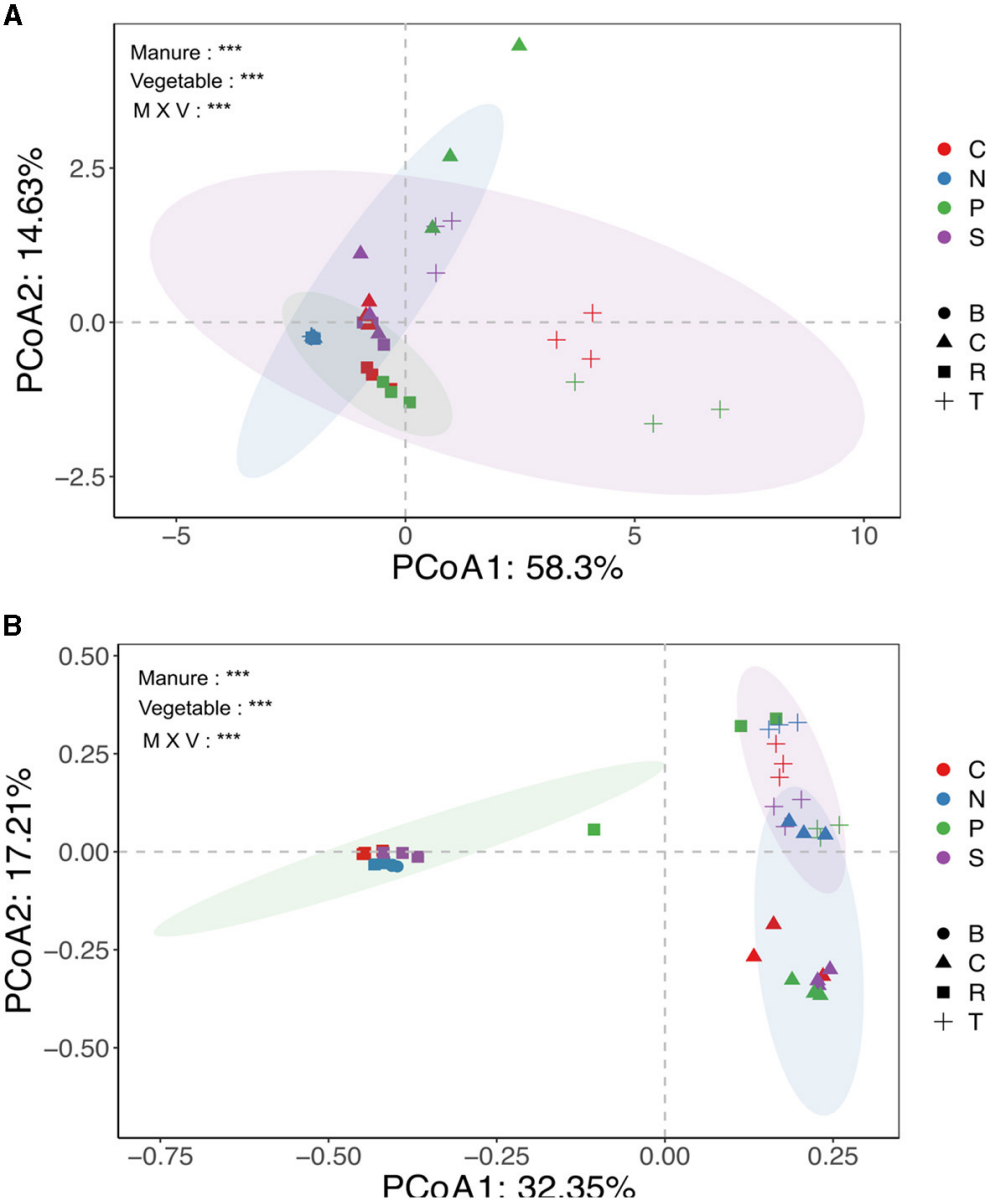
Principal coordinate analysis (PCoA) showing the overall distribution pattern of ARGs **(A)** and microbial communities **(B)** in different treatment groups. The different colors indicated fertilization sources, and the shapes showed vegetable types. N, C, P, and S represented no, chicken, pig, and sheep manures groups, respectively, while B, R, T, and C represented blank soil, carrots, tomatoes, and cucumber, respectively. Significant codes (^***^0.001 ^**^0.01 ^*^0.05) Adonis test.

The study detected and compared eight targeted antibiotic resistance genes (TRGs)—tetB, tetG, tetL, tetM, tetQ, tetW, tetX, and tet37—and an *intl1* gene across various treatment groups. Prior to the experiment, soil and manure compositions were analyzed, showing varying levels of TRG abundance. CM had the highest TRG abundance, followed by PM and SM, indicating that different manure types had different TRG profiles (Figure 1A). The absolute abundance of TRGs (copies g^−1^ dry weight) in the rhizosphere of different vegetables was significantly and variedly affected by manure amendments. Pig (P) and chicken (C) manure treatments notably affected TRG abundance, ranging from 5.2 × 10^∧^6 to 9.5 × 10^∧^8 and 8.0 × 10^∧^6 to 4.0 × 10^∧^8 copies per gram of soil, respectively. Sheep (S) manure had a lesser impact, ranging from 1.4 × 10^∧^6 to 3.8 × 10^∧^8 copies per gram of soil, still higher than that in the control (N) group. The *intl1* gene abundance significantly increased in manure-amended soils, except in the cucumber rhizosphere (Figure 1B). Among the vegetable types, TRG abundance was highest in the tomato rhizosphere, ranging from 2.0 × 10^6^ to 4.5 × 10^8^, followed by cucumbers (3.0 × 10^6^ to 3.9 × 10^8^), and the lowest abundance was observed in carrots (1.3 × 10^6^ to 6.9 × 10^7^) (Figure 1B). For instance, clustering analysis based on the Euclidean dissimilarity index showed significantly distinct distribution patterns of TRGs in both vegetable and manure groups (P = 0.001), along the first axis 58.3% and second 14.6 % variation (Adonis test, R^2^ = 0.26) (Figure 2A), indicating strong effects of vegetable and manure types on TRG acquisition in the rhizosphere. Specifically, TRGs in the tomato rhizosphere clustered distinctly from other vegetables, and pig manure groups clustered separately from other manure and control groups. These analyses supported the statement that in addition to different manure sources, the vegetable type also plays a significant role in the acquisition of TRGs and mobile genetic elements in the vegetable's rhizosphere. It emphasizes the importance of considering both factors in agricultural practices to mitigate potential environmental risks associated with antibiotic resistance.

### 3.3 Effects of manure and vegetable types on bacterial communities

The bacterial community comprised 10 dominant phyla with a relative abundance of > 1%, with Proteobacteria, Firmicutes, Bacteroidetes, Actinobacteria, and Cyanobacteria being the most abundant across all study groups. Pretreated soil exhibited a higher abundance of Proteobacteria (39.5%), Actinobacteria (30.8%), and Cyanobacteria (11.2%), while CM was predominantly enriched in Firmicutes (75.6%), Actinobacteria (9.7%), Bacteroidetes (9.7%), and Proteobacteria (4.4%). Similarly, SM was enriched in Proteobacteria (40.1%), Firmicutes (19.8%), Bacteroidetes (18.1%), and Actinobacteria (18.8%), while PM exhibited dominance in Firmicutes (57.7%), Proteobacteria (23.7%), and Bacteroidetes (4.1%) (Figure 1C).

Notably, Proteobacteria were dominant in the N treatment groups, particularly in NR (64%), NC (61%), and NT (49%) treatment group. Other phyla in the N treatment groups did not show significant differences compared to the BS. In manure-amended groups, a slight decrease in Proteobacteria was observed. Firmicutes exhibited a notable increase in the P treatment group (32%), followed by S (6.8%) and C (2.5%) treatments, compared to the N treatment group (0.52%). Chicken manure treated groups initially showed higher Firmicutes abundance, but the C treatment group showed the lowest abundance in Firmicutes, implying that Firmicutes in chicken manure had a lower survival rate as compared to other manures in rhizosphere soil. Bacteroidetes showed a non-significant increasing trend across treatment groups: N (8.9%), P (10%), S (17%), and C (30%), whereas, Actinobacteria abundance decreased in S (14.9%), P (8.6%), and C (8.3%) treatment groups as compared to N group levels (17.4%) (Figure 1D).

The PCoA based on Bray–Curtis distance revealed significant clustering of bacterial communities based on vegetable and manure types. The N treatment group exhibited distinct separation from manured groups, with different manure-treated and vegetable groups also clustering differently. Statistical analysis confirmed the significance of these clusters (R^2^ = 0.51; *p* = *0.001*) (Figure 2B). However, alpha diversity did not significantly differ among treatment groups (Supplementary Figure S3). The VPA indicated that bacterial communities contributed 12.2% to TRG variation (Figure 6B). Overall, these findings suggested that both manure and vegetable types significantly influence beta diversity, while having a minor impact on alpha diversity in rhizosphere bacterial communities.

### 3.4 OTUs distribution

**Figure 3:**
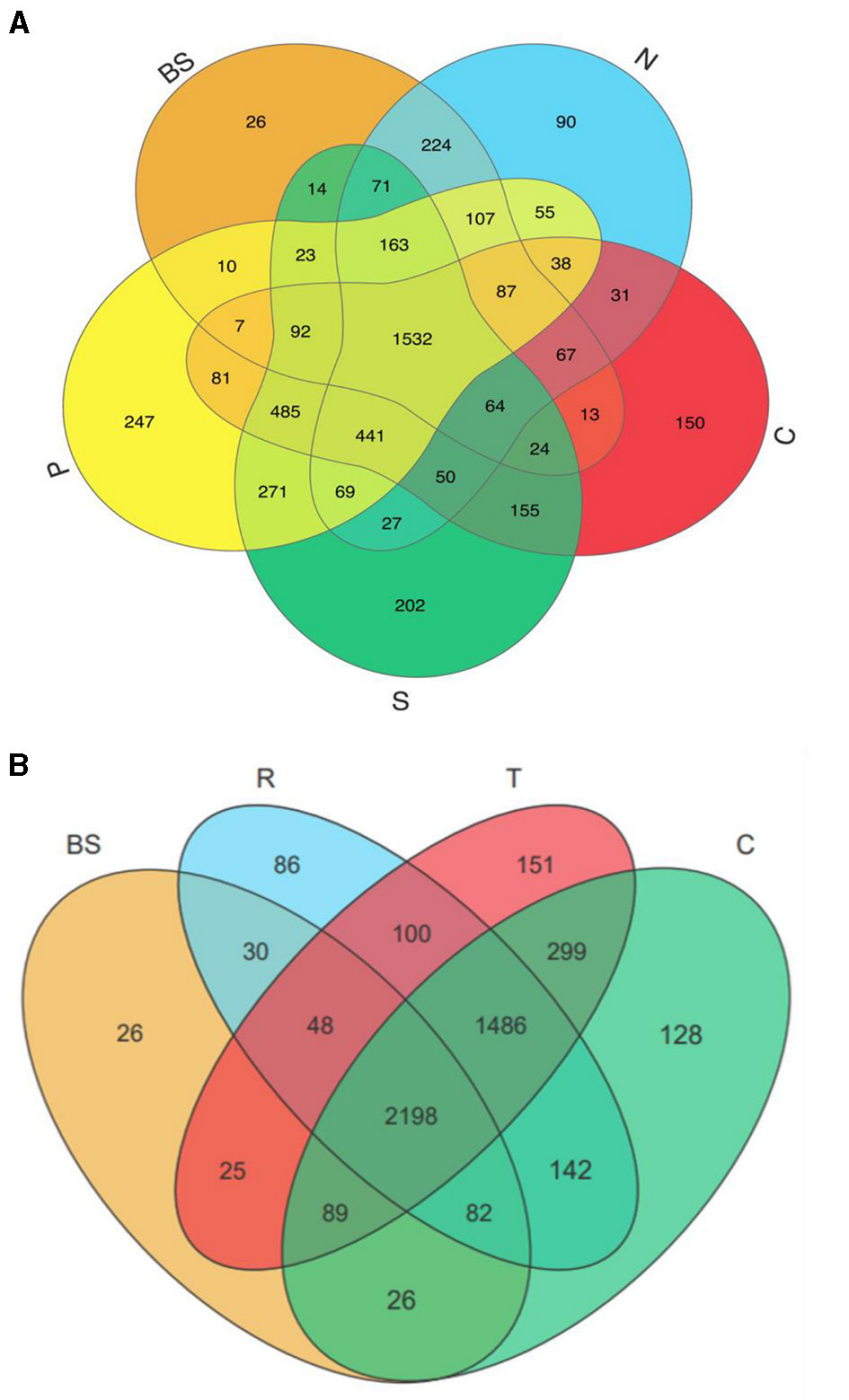
Venn diagram showing the bacterial OTUs numbers shared and unique by different treatments. BS, blank soil; N, no; C, chicken; S, sheep; P, pig manure treatment groups **(A)**. While **(B)** shows the shared and unique OUTs among blank and different vegetables in rhizosphere soil. BS, R, T, and C represented blank, carrots, tomatoes, and cucumber soil, respectively.

The distribution of operational taxonomic units (OTUs) among different vegetable and manure treatment groups revealed distinct patterns. P treatment groups exhibited the highest number of unique OTUs (247) and shared OTUs (3461), followed by S treatment groups with 202 unique and 3,481 shared OTUs, C treatment groups with 150 unique and 3,167 shared OTUs, N treatment groups with 90 unique and 3,026 shared OTUs, and the BS group with the lowest unique (26) and shared (2,585) OTUs (Figure 3A). Among vegetable treatment groups, tomatoes showed the highest number of OTUs, with 151 unique and 4,245 shared, followed by cucumbers with 128 unique and 4,322 shared, and carrots with 86 unique and 4,086 shared OTUs (Figure 3B). In contrast, the BS group had fewer than 26 unique and 2,498 shared OTUs. These findings suggest that manure can introduce a distinct microbial population and support the growth of endogenous bacteriomes in the soil. Additionally, different plant types significantly influenced soil microbiota, with a similar impact observed on the abundance of TRGs.

### 3.5 Correlation of TRGs with *intl1* gene, bacterial communities, and soil properties

**Figure 4:**
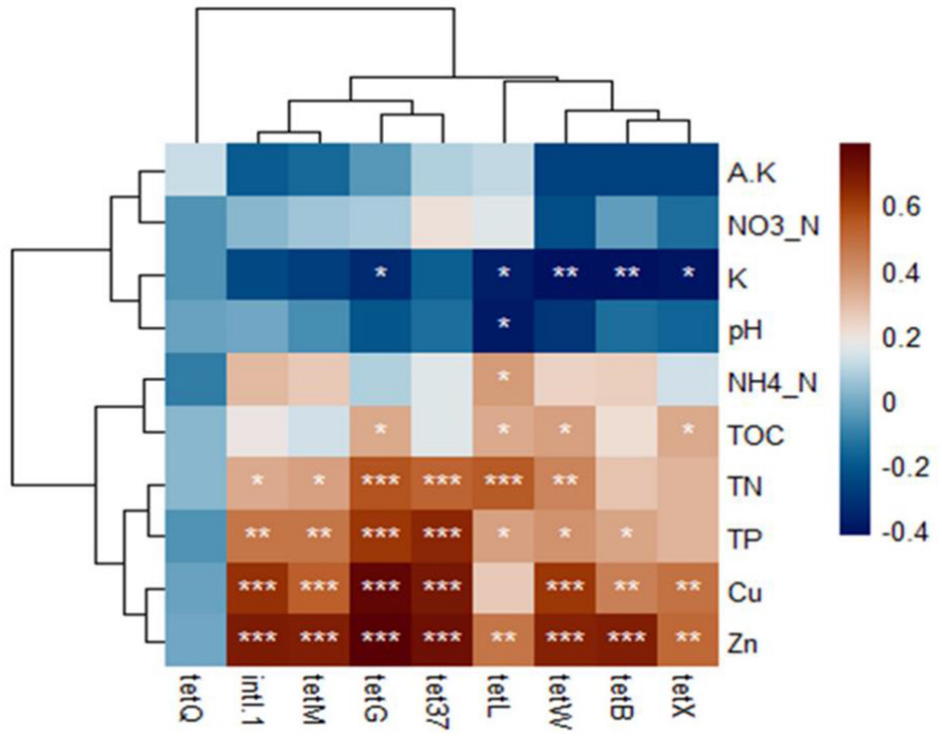
The Pearson's correlation among the soil properties, TRGs, and *intl1* gene. ^*^, ^**^, ^***^ indicated statistical significance levels of 0.05, 0.01, and 0.001, respectively. The displayed by p-heatmap. Dark brown and blue cells indicate positive and negative correlations, respectively.

Various factors influenced the distribution of TRGs in rhizosphere soil, including soil properties, heavy metals, and bacterial communities. Most selected TRGs exhibited significant positive correlations with heavy metals (Zn and Cu) and soil properties such as total phosphorus (TP), total nitrogen (TN), and total organic carbon (TOC) (Figure 4). However, the tetQ gene did not show significant correlations with these soil properties. Total potassium (K) displayed a significant negative correlation with most TRGs, whereas nitrate nitrogen (NO_3_-N) and available potassium (A.K) showed negative correlations with TRGs, although not statistically significant (*p* < 0.05). The study did not consider pH because vermiculites are used for augmenting vegetable growth in clay soil, which acts as pH buffering agents in soil (Indrasumunar and Gresshoff, 2013).

**Figure 5:**
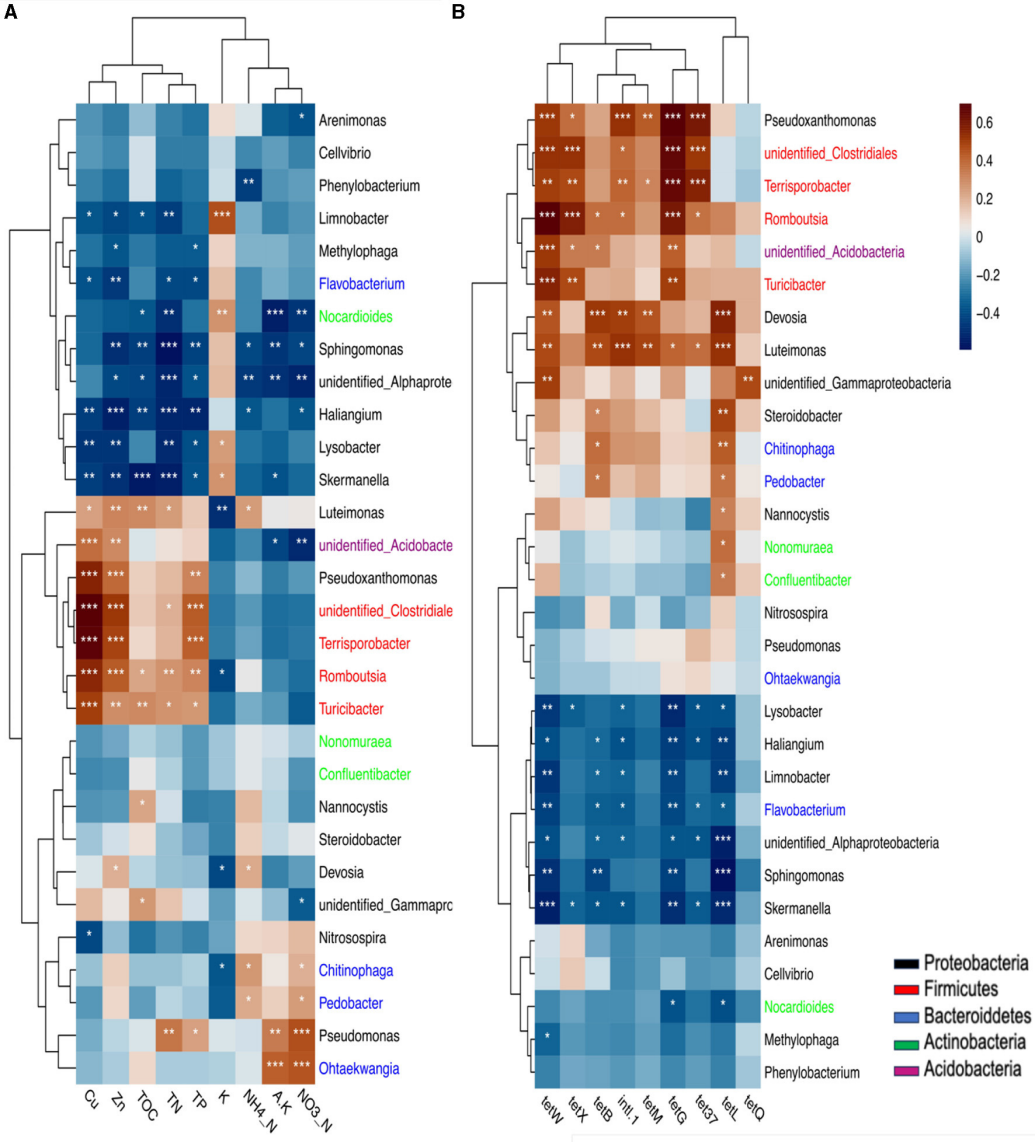
The correlation of soil properties and bacterial community at genus and phylum level **(A)**. the correlation of these communities with TRGs and *intl1* gene **(B)** based on Pearson's correlation is presented by P heat map, significant. codes: 0 “^***^” 0.001 “^**^” 0.01 “^*^”. The same color means the genus belongs to one phylum.

Furthermore, strong positive correlations were observed between the abundance of phylum Firmicutes, specifically with genera *Terrisporobacter, Romboutsia, Turicibacter*, and *unidentified Clostridiales*, and TP, TN, Zn, and Cu levels in the soil. Similarly, Proteobacteria genera like *Pseudoxanthomonas and Luteimonas* were positively correlated with these soil properties (Figure 5A). These genera had a positive correlation with soil properties and showed strong associations with TRGs and *intl1* genes. For instance, Firmicutes genera, including *Terrisporobacter, Romboutsia, Turicibacter*, and *unidentified Clostridiales*, exhibited robust positive correlations with *tetX, tetW, tetG, tet37*, and *intl1* genes, while Proteobacteria genera were significantly associated with various TRGs and *intl1* genes. Specifically, *Pseudoxanthomonas* was significantly associated with *tetG, tet37, TetW*, and *intl1* genes, and *Devosia* and *Luteimonas* genera were significantly associated with *tetL, tetM, tetB, tetW*, and *intl1* genes. Furthermore, the *tetQ and tetW* genes were significantly associated with *unidentified_Gamaprotobacteria* of Proteobacteria (Figure 5B).

Overall, Figure 5B shows that mostly Firmicutes and Proteobacteria were positively correlated with these genes. In contrast, *Gemmatimonadetes* and *Cyanobacteria* demonstrated a significantly negative *(p*<*0.05)* correlation with *tetG, tetW, and tetX*. Additionally, *Cyanobacteria* showed a significant *(p*<*0.05)* negative correlation with the *tetB* and *tetL* genes. Unidentified bacteria were negatively correlated with *tetW, tetG, and tetL (p*<*0.05)*. The phyla Actinobacteria and Choloroflexi were also negatively associated with the *tetG and tet37* genes (Supplementary Figure S3).

**Figure 6:**
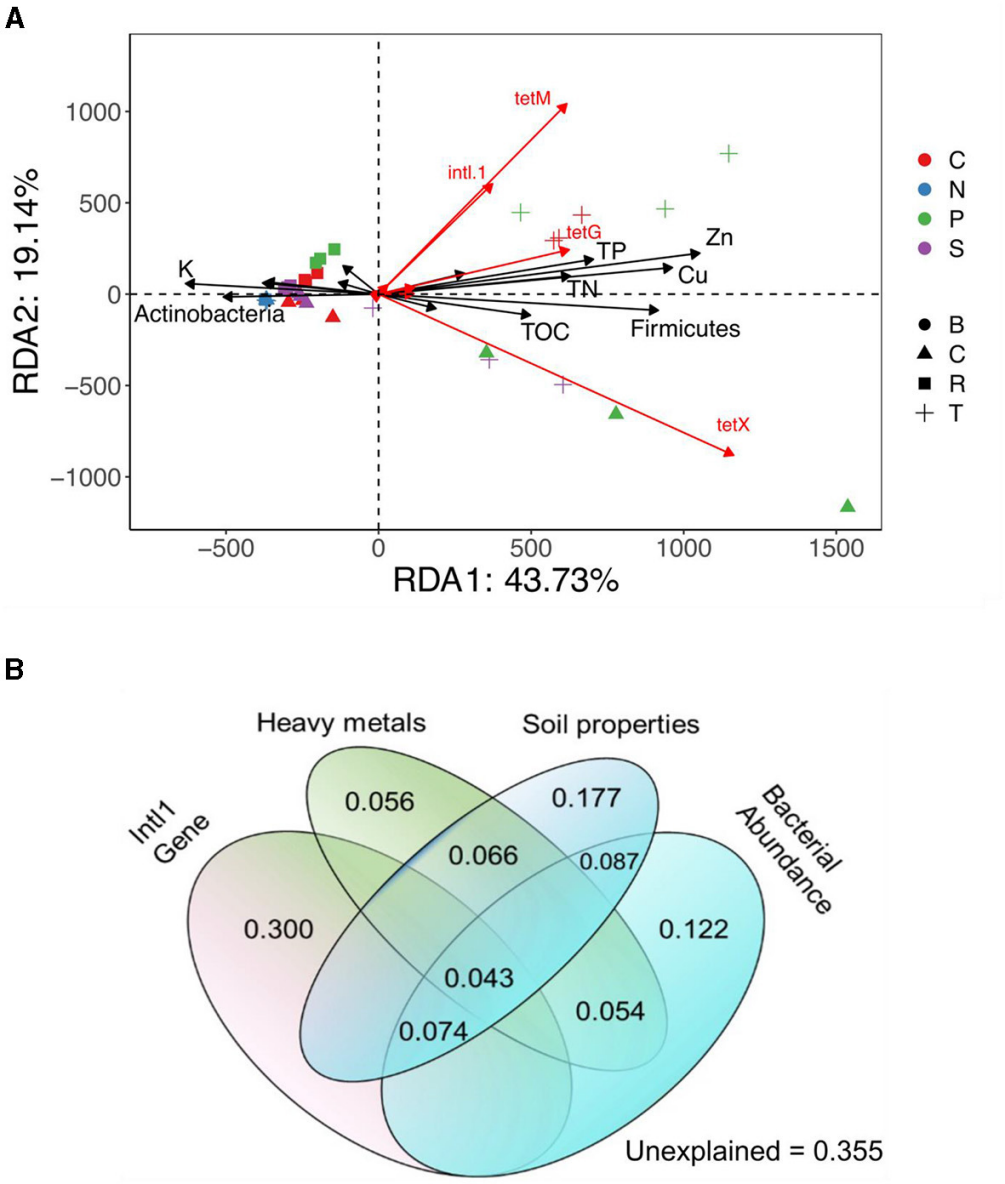
The contribution of soil properties and bacterial communities at the phylum level in the distribution profiles of TRGs in different Treatment Groups, presented by Redundancy analysis (RDA) **(A)**. Variation partitioning analysis (VPA) separates the contribution of soil properties, heavy metals, *intl1* gene, and bacterial community to the variation of TRGs **(B)**.

Redundancy analysis (RDA) further elucidated the relationship between bacterial community composition at the phylum level and TRG profiles, indicating significant effects of Firmicutes and *intl1* genes on TRG abundance. Soil properties (TN and TP) and heavy metals (Zn and Cu) were strongly related to TRG abundance (Figure 4A). The VPA revealed that the *intl1* gene contributed to 30% of the variance in TRGs, followed by soil properties (17.7%), bacterial communities (12.2%), and heavy metals (5.6%). Shared effects indicated that the soil properties (44.7%) and *intl1* gene (41.7%) were the most important factors explaining 86% of the variance in TRG profiles, while bacterial communities (38%) and heavy metals (21.9%) also contributed to variance in TRGs (Figure 6B).

## 4 Discussion

### 4.1 Effects of manures and vegetable type on TRGs and *intl1* gene

Our findings elucidate the factors influencing the colonization of resistant bacterial communities in the rhizosphere and their potential transfer to vegetables, posing a risk to human health. Previous studies have shown a significant overlap between plant and soil resistomes, indicating that soil is a primary source of plant-associated resistomes (Zhang et al., 2019; Yang et al., 2021). Consistent with former research (Wang et al., 2015; Guo et al., 2021), we observed that the abundance of TRGs and *intl1* genes was significantly higher in manure-treated soil compared to untreated soil. Moreover, different vegetable species grown in the same manure-amended soil selectively enriched distinct bacterial communities and TRGs. This supports previous findings that plant identity significantly influences the composition of tetracycline-resistant bacteria (TRBs), TRGs, and the *intl1* gene in rhizosphere soil (Chen et al., 2020; Guo et al., 2021). The variation in TRGs and *intl1* gene abundance might be attributed to the variation in soil organic matter provided by each vegetable, which determines the specific TRG-bearing bacterial community and offers hot spots for horizontal gene transfer (Cytryn, 2013; Chen et al., 2020; Zhang et al., 2020).

Our results revealed that pig manure-treated groups had significantly higher TRG abundance and diversity (*p* < 0.05), followed by chicken manure-treated groups, compared to sheep and no manure treatment groups (Figure 1A, Supplementary Table S3). This is consistent with the observations of previous studies, indicating that pig and poultry manures enhance the diversity and abundance of soil ARGs more than cattle manures (Zhang et al., 2017; Wang et al., 2019; Xu et al., 2020; Fu et al., 2021). The differences in animal diets, antibiotic use, and gut resistomes likely contribute to varying manure compositions and their effects on soil microbes. Additionally, pig and chicken manures contained higher levels of heavy metals, which were strongly correlated with TRGs and the *intl1* gene, echoing findings by Zhou et al. (2016) and Mazhar et al. (2021), that ARGs, including TRGs, were significantly associated with heavy metals in manures (Figure 4). These results underscore the importance of considering both manure type and vegetable species in agricultural practices to manage soil health and mitigate the dissemination of antibiotic resistance genes. Our findings highlighted that host identity is likely to be a critical driver of rhizosphere functions and composition, and evidence from prior research demonstrated that plants shape the rhizosphere microbial communities to their benefit and utilize their functional repertoires (Ling et al., 2022). Our results further revealed that the abundance of TRGs and *intl1* gene significantly increased with each type of manure, while vegetables recruited distinct TRGs and *intl1* gene abundance (Figure 1A; Supplementary Table S3). Additionally, PCoA further supports that different vegetables and manure types influence TRG composition differently (Figure 2A). Among the vegetable types, the tomato rhizosphere was significantly enriched in TRGs and the *intl1* gene. while the carrot's rhizosphere had notably fewer TRGs in all manure-treated groups. It is significant because the edible part of the carrot is near the rhizosphere, where soil and manure- associated TRBs may integrate into the carrot microbiome. The reduced TRG abundance in the carrot rhizosphere might be due to less diverse microbial community and single root or limited resources. Similarly, a couple of studies carried out earlier found fewer resistant genes in carrots grown in raw manure compared to leafy vegetables (Tien et al., 2017; Yang et al., 2018), whereas the high diversity and abundance of TRGs and the *intl1* gene in the tomato rhizosphere could result from different root exudates and an extensive below-ground fibrous root system that creates a hot spot for microbial colonization and competition. These findings help understand the varying degrees of risk among vegetables in recruiting TRGs from different manure types. The results underscore the need for proper treatment of widely used organic fertilizers, especially swine and poultry manures, to reduce the transmission of antibiotic resistance genes into the soil and ultimately into human bodies via vegetables and other crops.

### 4.2 Effects of manure and vegetable types on soil physiochemical properties and their correlation with TRGs

The soil properties are pivotal in shaping ARG patterns in different cropland fields (Tiedje et al., 2019; He et al., 2021). Previous studies have shown that manure fertilization can alter soil properties and enrich the TRBs (Wang et al., 2018; Wu et al., 2021). These studies indicate that the type of plants and manures significantly influence soil properties. Our results showed that pig and chicken manure significantly increased most soil properties compared to sheep and the untreated groups. Specifically, application of the pig manure significantly increased levels of heavy metals such as Cu and Zn, as well as TN, TP, and TOC in the tomato rhizosphere (Table 1). The enhancement of these soil factors may explain the increased acquisition of TRGs in the tomato rhizosphere. We also observed that the *intl1* gene, which contributed to 41.3% of TRG variance, had a strong positive correlation with Cu, Zn, TP, and TN, which might explain the higher mobilization of TRGs within bacterial communities. Compared to carrots and cucumbers, tomatoes have high root proliferation and biomass, along with the release of various photosynthates, which may modify soil properties favorably for TRG-containing bacteria. Variance partitioning analysis (VPA) showed the importance of soil properties and heavy metals, explaining 44.7% and 21.9% of the variance in TRG profiles, respectively (Figures 6A, B). These results confirm the significant impact of soil properties on TRGs and *intl1* genes. Similarly, a higher concentration and positive correlation of Cu, Zn, TN, SOC, and TP with ARG abundance in pig and layer-manured fields were found by Sui et al. (2019) and Wang et al. (2020). Another study stated that crops could shape the overall rhizosphere consistently through the secretion of various exudates (Bulgarelli et al., 2013). The addition of Cu and Zn in pig and poultry feed for intensive production also leads to higher levels of these heavy metals in the manure and soil, which may exert selection pressure for TRGs. The positive correlation between the soil factors and TRGs indicates that these factors influence the selective recruitment and enrichment of bacteria containing TRGs. Our analysis did not include soil pH due to the use of vermiculites as a buffering agent, as stated by Indrasumunar and Gresshoff (2013). Vermiculite's buffering capacity makes it unsuitable for studying the effect of soil acidity. Cu, Zn, TP, and TN were positively correlated with most of the targeted TRGs and *intl1*gene, except for *tetQ*. Similarly, Wang F. et al. (2022) and Li et al. (2022) found that Cu, Zn, pH, TN, SOC, and TP significantly influenced bacterial diversity, composition, richness, and root biomass, which directly affect ARG abundance. Additionally, *tetG, tetL, tetW, tetB*, and *tetX* were negatively correlated with potassium (K) (Figure 4). The negative correlation between potassium and TRGs suggests that further research is needed to explore the impact of potassium on ARGs. Despite these findings, it is essential to acknowledge that the agricultural field is more complex than controlled pot experiments. Field-based experiments on more vegetables, including those with edible parts grown both above and below ground, are necessary to assess and mitigate the risk of ARGs comprehensively.

### 4.3 Rhizo-bacteriome recruitment by various vegetables and their correlation with TRGs

The diversity and composition of the rhizospheric bacterial community are functions of both plant species and soil properties (Vorholt et al., 2017; Guo et al., 2021). Plants skillfully utilize their resources and shape rhizobiome to their benefit. The rhizosphere is a hot spot involving interactions between manure-associated and indigenous microbial communities, forming a distinct community and sharing various resistance genes (Ling et al., 2022). Organic fertilizers, such as manures, introduce manure-associated bacteria and selectively nourish the growth of indigenous soil bacteria that may harbor ARG-carrying bacteria and also facilitate HGT of ARGs to soil indigenous bacteria (Smalla et al., 2001; Hu et al., 2016; Zhang et al., 2020). Similarly, our study investigated how plant and manure types influence the structure of soil bacterial communities, leading to shifts in TRG profiles. The results showed that each plant type acquired a distinct bacterial community. For example, the tomato rhizosphere was enriched with unique and shared OTUs compared to other plants (Figure 3B). This finding was further confirmed by redundancy analysis, which showed the association between bacterial communities and TRGs. Variance partitioning analysis revealed the overall role of the bacterial community (38%) and *intl1* gene (41.7%) in the variance of the TRG profile. Consistently, previous studies have suggested that different plants have preferences for specific microorganisms due to differences in physicochemical properties, bacterial community, and mobile genetic elements, which are the main factors affecting the ARG distribution (Chen et al., 2017; Zhou et al., 2019). Additionally, principal coordinate analysis based on the Bray–Curtis dissimilarity index and Euclidian distance showed that both manure and plant type significantly impact the diversity of the bacterial community and TRGs. Among the four most abundant phyla in manures and manure-treated groups, some genera of Firmicutes and Proteobacteria were strongly correlated with TRGs and may be the potential bacterial hosts carrying TRGs (Figure 5B). These bacterial phyla have been recognized as important hosts for multiple ARGs in the metagenomics analysis (Forsberg et al., 2012; Han et al., 2018; Zhao et al., 2020; Ellabaan et al., 2021; Wu et al., 2022). Pig manure treatments harbored higher levels of Firmicutes and Proteobacteria, which correlated with the presence of targeted ARGs and the *intl1* gene. In contrast, chicken manure treatments exhibited higher levels of Proteobacteria associated with these genes. The study identified potential bacterial genera from Firmicutes and Proteobacteria as hosts for the targeted ARGs and mobile genetic elements (Figure 1B). Genera belonging to phylum Firmicutes, including *Terrisporobacter, Romboutsia, Turicibacter*, and *unidentified_Clostridiales*, and those in phylum Proteobacteria, such as *Pseudoxanthomonas* and *Luteimonas* showed a strong positive correlation with TP, TN, Zn, and Cu. According to our findings, these genera were significantly correlated with TRGs and the *intl1* gene, indicating that soil properties indirectly influence TRGs. Likewise, multiple studies have demonstrated that plants and manures shape soil microbial communities by providing nutrients, such as root exudates, or by altering soil properties (Chen et al., 2017; Wang et al., 2019, 2020; Zhang et al., 2019; Pu et al., 2020).

Our findings elucidate the factors influencing the rhizosphere bacteriome and the presence of tetracycline resistance genes (TRGs) in agricultural systems. This study highlights the diverse effects of different vegetables on the acquisition of TRGs and *intl1* gene in manure-amended soils. The results confirm that manure types have varying impacts on TRGs, soil properties, and bacterial communities. Additionally, the vegetable type significantly shapes the soil microbiome and properties, exerting selective pressure on TRGs, the *intl1* gene, and associated bacteria. We identified the possible potential host genera for these TRGs and mobile genetic elements within bacterial communities.

However, our study has several limitations, such as focusing only on targeted tetracycline resistance genes and pot experiments, not actual agricultural fields. Further study is needed to assess the role of plant identity and anthropogenic disturbances and identify risks associated with different vegetables. To obtain more precise and quantifiable results, further attention and approaches to this area of study are needed, which will help develop strategies for controlling the dissemination of ARGs in One-health sectors.

## 5 Conclusions

Our research highlights the significant impact of pig and chicken manures on the presence and abundance of TRGs and the *intl1* gene in rhizosphere soil, likely influenced by the animals' diets and species. These manures contain distinct bacterial communities and nutrient compositions, which exert specific selective pressures on soil bacterial populations. Additionally, our findings indicate that different vegetable types influence the composition of their rhizosphere's resistome. For instance, tomatoes were found to recruit a higher abundance of TRGs and the *intl1* gene across all manure types. This may be attributed to their extensive fibrous root systems that provide more energy and carbon sources, thereby attracting diverse bacterial communities with elevated TRG and *intl1* gene levels. Therefore, it is crucial to understand the role of different vegetable types in the acquisition of TRGs in the rhizosphere, which serves as a bridge in the One Health sector—linking animal and plant resistomes and subsequently propagating to humans. Despite these findings, further research is required to examine the full spectrum of antibiotic resistance genes (ARGs) and a broader range of vegetables in actual agricultural fields, which present greater complexity than controlled pot experiments. Identifying vegetables and manure types with varying levels of risk is essential for developing comprehensive risk assessment strategies aimed at mitigating the dissemination of resistance genes across one health component (plant, animal, human, and ecosystem).

## Data availability statement

The datasets presented in this study can be found in online repositories. The names of the repository/repositories and accession number(s) can be found in the article/Supplementary material.

## Author contributions

IzA: Conceptualization, Investigation, Methodology, Visualization, Writing – original draft. BN: Writing – review & editing, Investigation. NA: Writing – review & editing, Investigation. ZL: Software, Visualization, Writing – review & editing. ZY: Investigation, Writing – review & editing. JC: Investigation, Writing – review & editing. KA: Writing – review & editing, Project administration, Funding acquisition. AM: Writing – review & editing, Project administration, Funding acquisition. IkA: Writing – review & editing. SF: Writing – review & editing. LS: Resources, Writing – review & editing. SX: Supervision, Conceptualization, Resources, Funding acquisition, Methodology, Project administration, Software, Visualization, Writing – review & editing. SC: Supervision, Conceptualization, Resources, Funding acquisition, Methodology, Project administration, Software, Visualization, Writing – review & editing.
