# Supplementary material for: Interplay among manures, vegetable types, and tetracycline resistance genes in rhizosphere microbiome
Source: Front Microbiol. 2024 Jul 1;15:1392789. doi: 10.3389/fmicb.2024.1392789 (PMC11246966; doi:10.3389/fmicb.2024.1392789)
Supplement: Supplementary file 1 [file Data_Sheet_1.docx]

***Supporting information***

**Interplay Among Manures, Vegetable Types, and Tetracycline Resistance Genes in Rhizosphere Microbiome**

**Fig. S1** Schematic of experimental design.

**
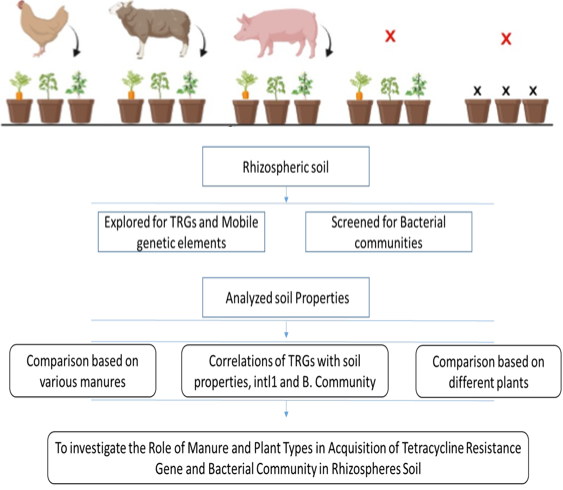
**

**Table S1.** Basic properties of soil, Chicken, Sheep and Pigs manures used in the pot experiment.

|  | Soil | Chicken manure | Sheep manure | Pigs manure |
| --- | --- | --- | --- | --- |
| Texture | Clay | - | - | - |
| pH | 7.5 | - | - | - |
| TP(mg/L) | 0.62 | 9.8 | 6.54 | 9.94 |
| TN/(mg/L) | 0.06 | 17.82 | 16.65 | 16.2 |
| K（g/kg) | 23.14 | 16.6002656 | 15.82633053 | 7.15835141 |
| Zn（mg/kg) | 50.84 | 16.60 | 15.83 | 650.84 |
| Cu（mg/kg) | 17.7 | 21.76 | 7.09 | 467.00 |
| Available K（mg/kg) | 121.34 | - | - | - |
| TOC(g/kg) | 4.16 | - | - | - |
| NH4_N(mg/L) | 0.19 | - | - | - |
| NO3_N(mg/L) | 0.44 | - | - | - |

Abbreviation: Cu, Copper; Zn, Zinc; K, total Potassium; A.K, Available Potassium; TN, total nitrogen; TP, total phosphorus; NO3_N, Nitrates; NH4_N, Ammonium and TOC, Total organic carbon.

**Table S2.** The primer sequences and details for the targeted TRGs and their resistance mechanism.

| Genes Name | Primers | Sequence (5’-3’) | Annealing Temperature (°C) | Amplicon  size (bp) | Resistance Mechanism | Reference |
| --- | --- | --- | --- | --- | --- | --- |
| 16S rRNA | **Forward Primer** | GGACTACGGATTAGATACCCTGGTAGTCC | 60 | - | - | Caporaso et al., 2011). |
|  | **Reverse Primer** | CTTGTGCGGGTCCCCGTCAAT |  |  |  |  |
| *tet*A | ***tet*A-FW** | GCGCGATCTGGTTCACTCG | 61 | 164 | Efflux | Aminov, R. I. *et al* 2002 |
|  | ***tet*A-RV** | AGTCGACAGYRGCGCCGGC |  |  |  |  |
| tetB | **tetB-FW** | TACGTGAATTTATTGCTTCGG | 59 | 206 | Efflux | Nemec et al., 2004 |
|  | **tetB-RV** | ATACAGCATCCAAAGCGCAC |  |  |  |  |
| tetC | ***tetC-FW*** | GCGGGATATCGTCCATTCCG | 68 | 207 | Efflux | Aminov et al., 2001 |
|  | ***tetC-FR*** | GCGTAGAGGATCCACAGGACG |  |  |  |  |
| *tet*G | ***tet*G-FW** | GCAGAGCAGGTCGCTGG | 65 | 134 | Efflux | CHEN, L. *et al* 2013 |
|  | ***tet*G-RV** | CCYGCAAGAGAAGCCAGAAG |  |  |  |  |
| tetL | **tetL-FW** | TCGTTAGCGTGCTGTCATTC | 55 | 267 | Efflux | Ng Martin *et al*., 2001 |
|  | **tetL-RV** | GTATCCCACCAATGTAGCCG |  |  |  |  |
| *tet*M | ***tet*M-FW** | ACAGAAAGCTTATTATATAAC | 55 | 171 | Ribosomal protection | Aminov, R. I., *et.,al* 2001 |
|  | ***tet*M-RV** | TGGCGTGTCTATGATGTTCAC |  |  |  |  |
| tetQ | ***tetQ-FW*** | AGAATCTGCTGTTTGCCAGTG | 56 | 169 | Ribosomal  protection | Aminov *et al*., 2001 |
|  | ***tetQ-RV*** | CGGAGTGTCAATGATATTGCA |  |  |  |  |
| *tet*W | ***tet*W-FW** | GAGAGCCTGCTATATGCCAGC | 64 | 168 | Ribosomal  protection | Aminov, R. I., *et.,al* 2001 |
|  | ***tet*W-RV** | GGGCGTATCCACAATGTTAAC |  |  |  |  |
| *tet*X | ***tet*X-FW** | GAAAGAGACAACGACCGAGAG | 56.5 | 131 | Antibiotic inactivation | Speer, B. S. *et.,al* 1992 |
|  | ***tet*X-RV** | ACACCCATTGGTAAGGCTAAG |  |  |  |  |
| Tet37 | **tet37-FW** | ATGGTTCGCTATTACTCTAAC | 45 | 177 | Antibiotic inactivation | Collins *et al.,* 2016 |
|  | **tet37-FW** | ATCAGTCTCATATTTCGACA |  |  |  |  |

**Table S3.** Effects of different manures, vegetables, and their interaction relative percentage of TRGs. The lowercase letters represent variations among treatment groups; bold values indicate significant differences *p < 0.5* (Tukey HSD Test). Meanwhile, the p-value and stars showed the overall effects of manures, vegetables, and their interaction. Significance Codes: 0 ‘***’ 0.001 ‘**’ 0.01 ‘*’ 0.05 ‘.’ (two-way permutation test).

| **Treatments** | **intl1%** | **tetB%** | **tetG%** | **tetL%** | **tetM%** | | **tetQ%** | **tetW%** | **tetX%** | **tet37%** |
| --- | --- | --- | --- | --- | --- | --- | --- | --- | --- | --- |
| **BS** | 0.17f | 0.18c | 0.05d | 0.29d | 0.00b | | 0.00a | 0.00d | 0.01b | 0.00b |
|  |  |  |  |  |  | |  |  |  |  |
| **NR** | 0.00f | 0.00c | 0.08d | 0.55cd | 0.00b | | 0.00a | 0.00d | 0.00b | 0.00b |
| **CR** | 1.17ef | 0.00c | 3.08cd | 2.77bcd | 1.44b | | 1.5a | 0.28d | 0.00b | 5.18b |
| **SR** | **1.89de** | 0.00c | 1.23d | 3.43abcd | 0.45b | | 1.44a | **3.58bc** | 0.00b | 0.41b |
| **PR** | **5.92c** | 0.00c | 2.01cd | 0.99cd | 2.67b | | 1.09a | 1.84cd | 0.00b | 5.24b |
|  |  |  |  |  |  | |  |  |  |  |
| **NT** | 0.00f | 0.00c | 0.02d | 0.25d | 0.00b | | 0.49a | 0.00d | 0.06b | 0.00b |
| **CT** | **9.18b** | **12.64a** | **4.46bc** | 6.73a | **12.18a** | | 4.87a | 4.61abc | 5.67b | 4.72b |
| **ST** | **2.66d** | **4.72b** | 2.21cd | 4.07abc | 1.47b | | 3.83a | 5.57ab | 7.07ab | 0.19b |
| **PT** | **11.80a** | **6.59b** | **9.47a** | 6.00ab | **14.73a** | | 2.78a | **7.03a** | 5.55b | **13.95a** |
|  |  |  |  |  |  | |  |  |  |  |
| **NC** | 0.00f | 0.00c | 0.09d | 0.55cd | 0.03b | | 0.51a | 0.00d | 0.04b | 0.00b |
| **CC** | 0.00f | 1.57c | 1.80cd | 2.77bcd | 0.00b | | 7.09a | 2.21cd | 1.05b | 1.78b |
| **SC** | 0.41f | 1.05c | 1.99cd | 3.98abc | 0.31b | | 6.78.5a | 2.08cd | 0.59b | 0.13b |
| **PC** | 0.15f | **6.59b** | **6.84ab** | 0.99cd | 0.06b | | 2.93a | **6.13ab** | **13.30a** | 1.73b |
|  |  |  |  |  |  |  | |  |  |  |
| **Vegetables** | <2e-16 *** | <2e-16 *** | <2e-16 *** | <2e-16 *** | <2e-16 *** | | 0.061 | <2.2e-16 *** | <2e-16 *** | <2e-16 *** |
| **Manures** | <2e-16 *** | <2e-16 *** | <2e-16 *** | <2e-16 *** | <2e-16 *** | | 0.146 | <2.2e-16 *** | <2e-16 *** | <2e-16 *** |
| **V*M** | <2e-16 *** | <2e-16 *** | <2e-16 *** | 0.0046 ** | <2e-16 *** | | 0.362 | 0.002** | <2e-16 *** | <2e-16 *** |

Abbreviations: The first letters B, N, C, S, and P represented blank, no, chicken, sheep, and pig manures, respectively; the second letters S, R, T, and C represented, blank, carrots, tomatoes, and cucumber, respectively. Significant. Codes: 0 ‘***’ 0.001 ‘**’ 0.01 ‘*’ and 0.05 ‘.’ (two-way permutation test) and small letters displayed Tukey HSD results.

**Fig. S2.** Relative abundance of bacterial community before treatment at genus level (B) and bacterial community at genus level in each treatment group Abbreviations: BS: Blank soil; CM: chicken manure; SM: Sheep manure; PM: Pig manure; the first letter N, C, S and P represented No manure, chicken manure, sheep manure and pig manure, and second letter R, T, and C denoted plant types, carrots, tomatoes, and cucumbers respectively.


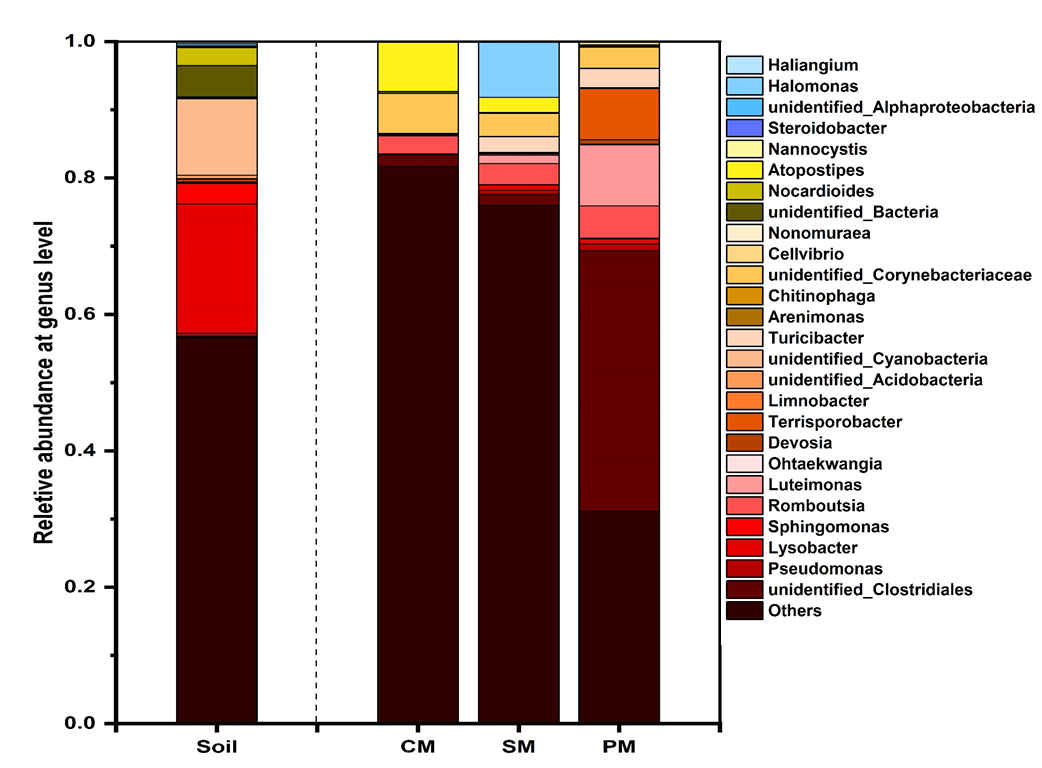

**Fig. S3.** Shannon Diversity Index


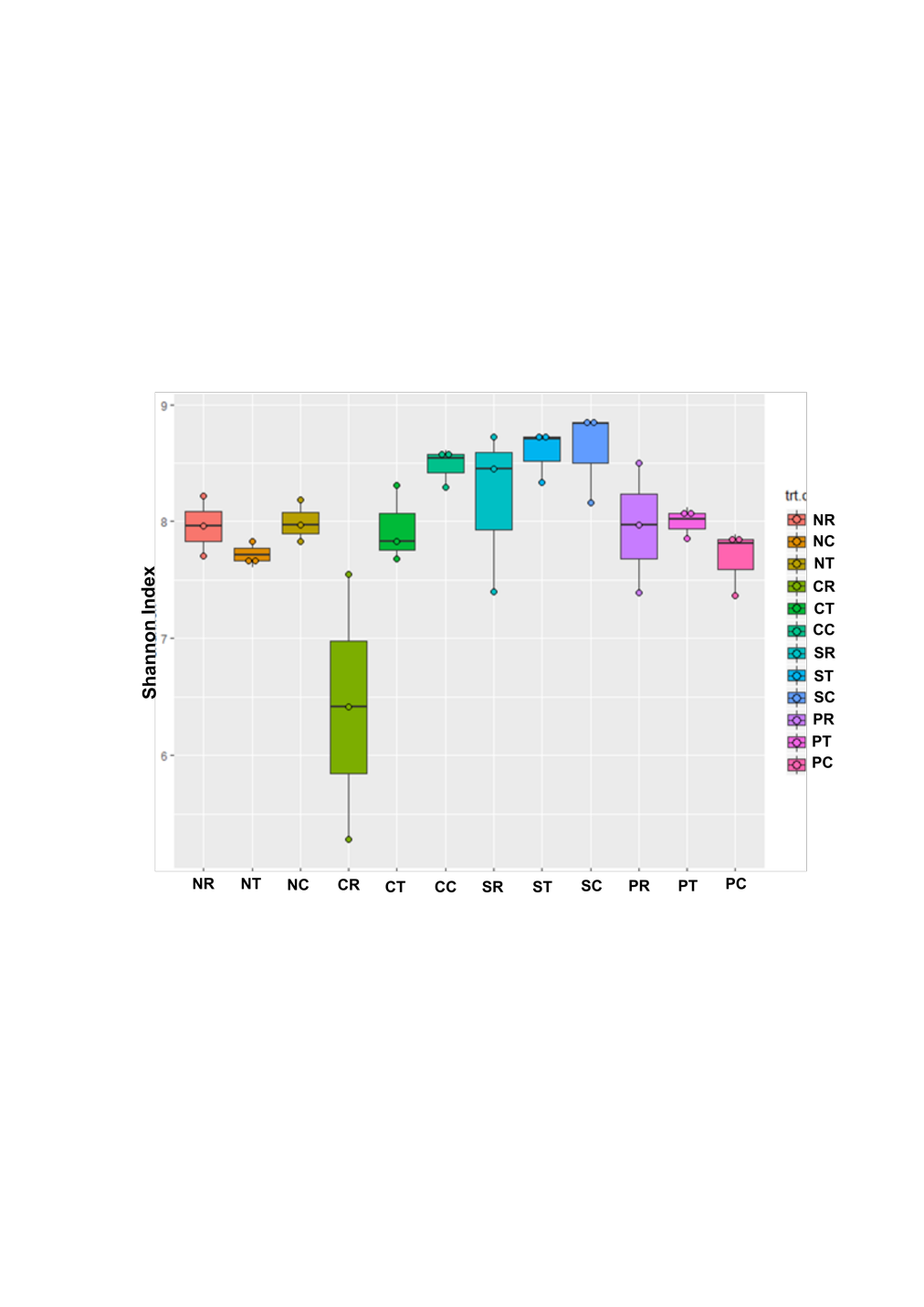


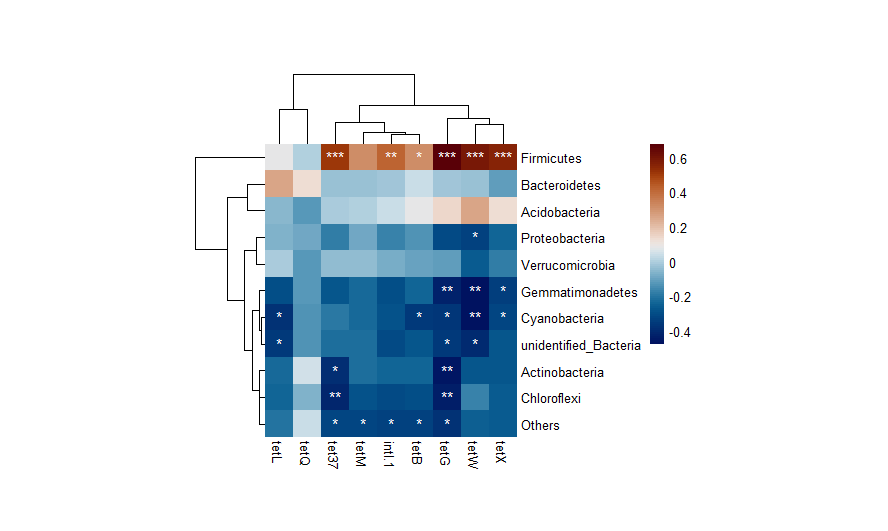


**Fig S3**. Correlation between TRGs and bacterial communities at phylum level
